# Supplementary material for: Niclosamide targets the dynamic progression of macrophages for the resolution of endometriosis in a mouse model
Source: Commun Biol. 2022 Nov 11;5:1225. doi: 10.1038/s42003-022-04211-0 (PMC9652344; doi:10.1038/s42003-022-04211-0)
Supplement: Supplementary file 2 — Supplementary Materials [file 42003_2022_4211_MOESM2_ESM.pdf]

Supplementary Information

Niclosamide targets the dynamic progression of macrophages for the resolution of endometriosis in a mouse model

Liang Zhao[1], Mingxin Shi[1], Sarayut Winuthayanon[1], James A. MacLean II[1], Kanako Hayashi[1,2]

[1]School of Molecular Biosciences, Center for Reproductive Biology, Washington State University, Pullman, Washington 99164, USA

[2]Correspondence to Kanako Hayashi: k.hayashi@wsu.edu

List of captions

Supplementary Figures (1-8)

Supplementary Data Legends (1-7)

Supplementary Figures

Supplementary Fig. 1: Characteristics of immune cells at the single-cell level.

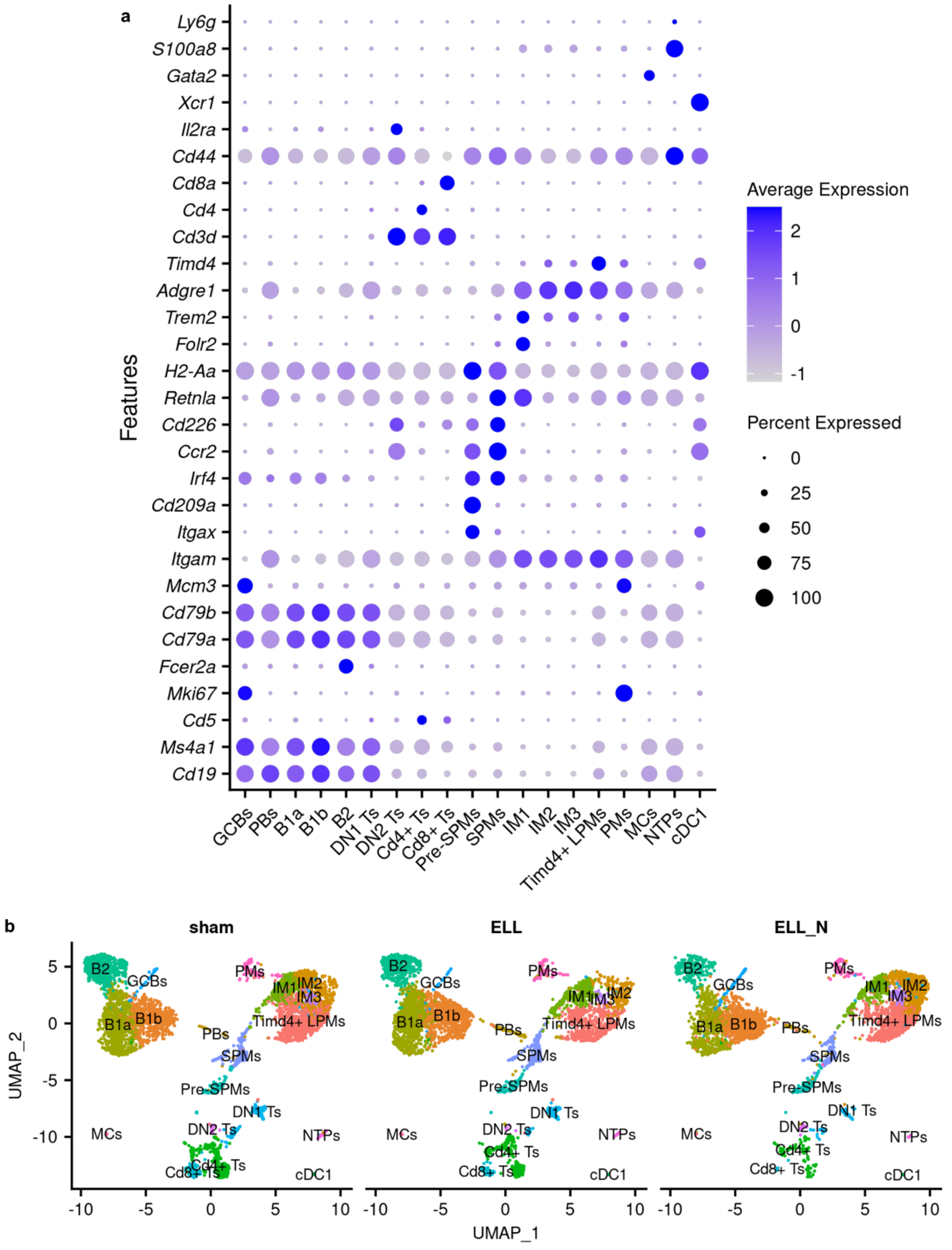

a Characteristic lineage-specific marker genes used to distinguish different cell subtypes. Note: expression levels are shown by "-" for none, "+' for low, "++" for intermediate, and "+++" for high. **b** UMAP distribution of cell populations within

three groups. Sham, sham control; ELL, endometriosis-like lesions; ELL_N, niclosamide administration to ELL-induced mouse; Pre-SPMs, premature “small” peritoneal macrophages; SPMs, “small” peritoneal macrophages; IM1-3, intermediate macrophages subtype 1-3; *Timd4*$^{+}$ LPMs, *Timd4*$^{+}$ “large” peritoneal macrophages; PMs, proliferating macrophages; B1a, B1a cells; B1b, B1b cells; B2, B2 cells; GCBs, germinal-center B cells; PBs, plasma blast B cells; *Cd4*$^{+}$ Ts, *Cd*4^{+} T cells; *Cd8*$^{+}$ Ts, *Cd8*$^{+}$ T cells; DN1, double negative T cells 1; DN2, double negative T cells 2; cDC1, conventional dendritic cells 1; MCs, Mast cells; NTPs, neutrophils.

Supplementary Fig. 2: Enriched GO terms of biological processes by the top 100 expressed genes within each macrophage subpopulation to show their characteristic profiles.

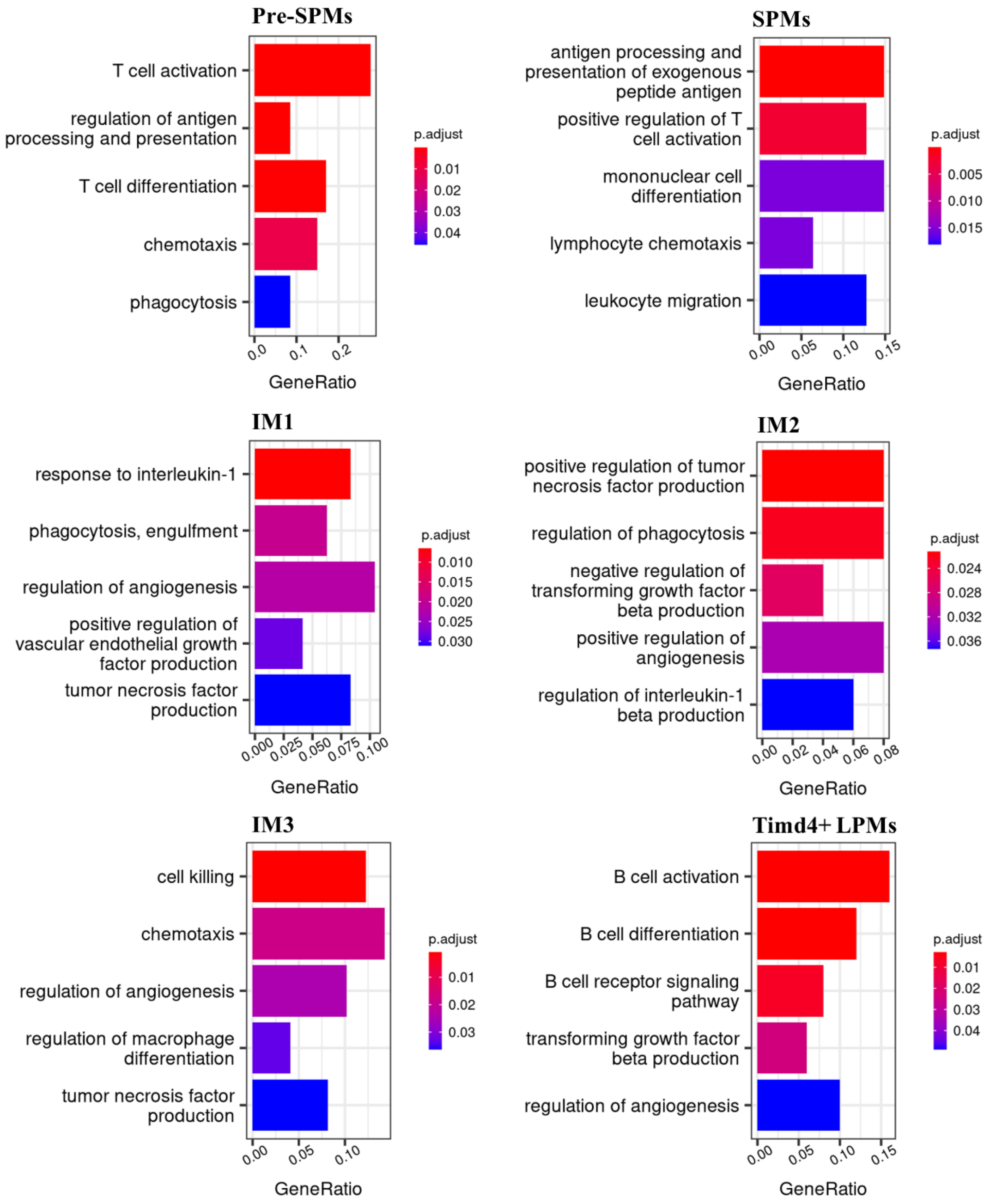

Pre-SPMs, monocyte-derived dendritic cells; SPMs, “small” peritoneal macrophages; IM1-3, intermediate macrophages subtype 1-3; *Timd4*$^{+}$ LPMs, *Timd4*$^{+}$ “large” peritoneal macrophages.

Supplementary Fig. 3: Re-analysis of a single-cell transcriptomic dataset of peritoneal macrophages in female mice in a normal physiological state from a public resource.

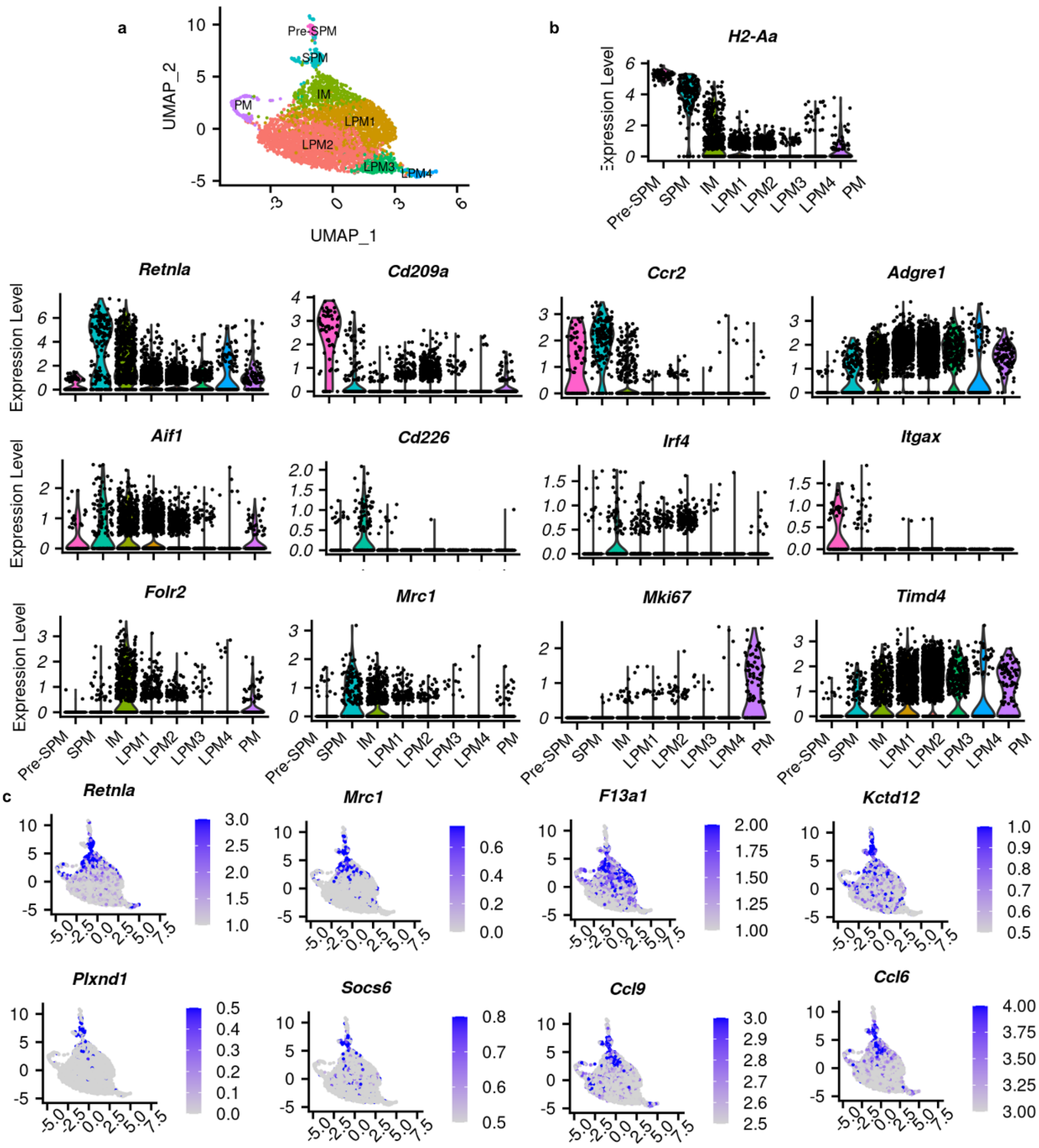

a UMAP visualization of macrophage populations. **b** Representative gene markers used for macrophage subpopulation clustering. **c** The featured plot showing the distribution of genes. Pre-SPM, premature "small" peritoneal macrophages; SPMs, "small" peritoneal macrophages; IM, intermediate macrophages; LPM1-4, *Timd4*$^{+}$ "large" peritoneal macrophage subtypes 1-4; PMs, proliferating macrophages.

Supplementary Fig. 4: The changes of peritoneal macrophage subpopulations with time after ELL induction in mice.

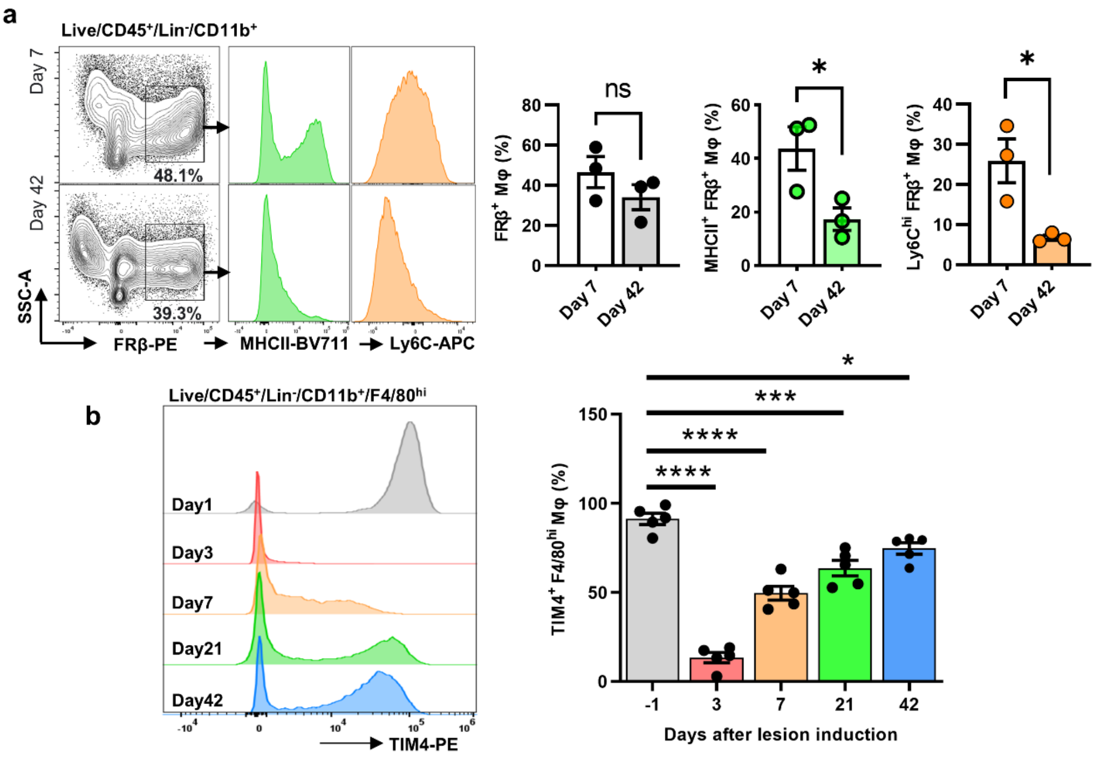

a Quantification of the MHCII^{+} or Ly6C^{high} subsets of CD11b^{+} FRβ^{+} macrophages within the peritoneal fluid on day 7 and 42 after ELL induction. n = 3 per group. **b** Dynamic changes of CD11b^{+} F4/80^{high} TIM4^{+} macrophages before (-1 day) and after ELL induction (day 3-42). $*p < 0.05$, $***p < 0.001$, $****p < 0.0001$. mean ± SEM, n = 5 per group.

Supplementary Fig. 5: Reconstruction of a trajectory path for the early maturation and differentiation of recruited macrophages (data from a public resource).

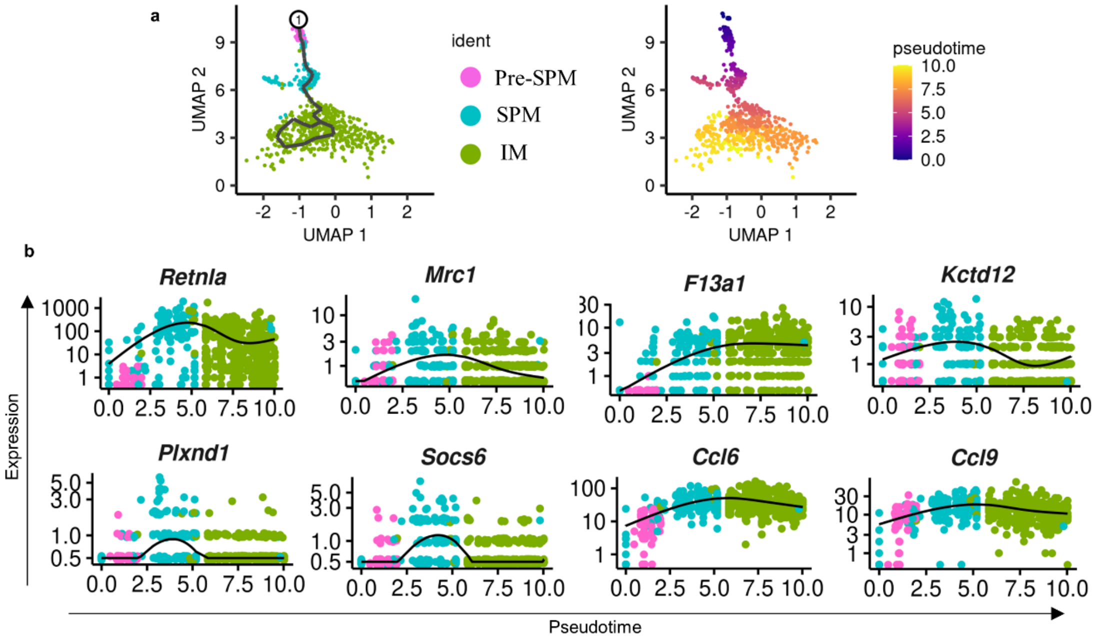

a UMAP showing the selected cells (Pre-SPM, SPM, and IM) and the trajectory path built within them. The selected root cell was labeled as ①. **b** Dynamic expression of genes along this trajectory path. Pre-SPMs, premature “small” peritoneal macrophages; SPM, “small” peritoneal macrophages; IM, intermediate macrophages.

Supplementary Fig. 6: Reconstruction of a trajectory path for Timd4+ “large” peritoneal macrophages (data from a public resource).

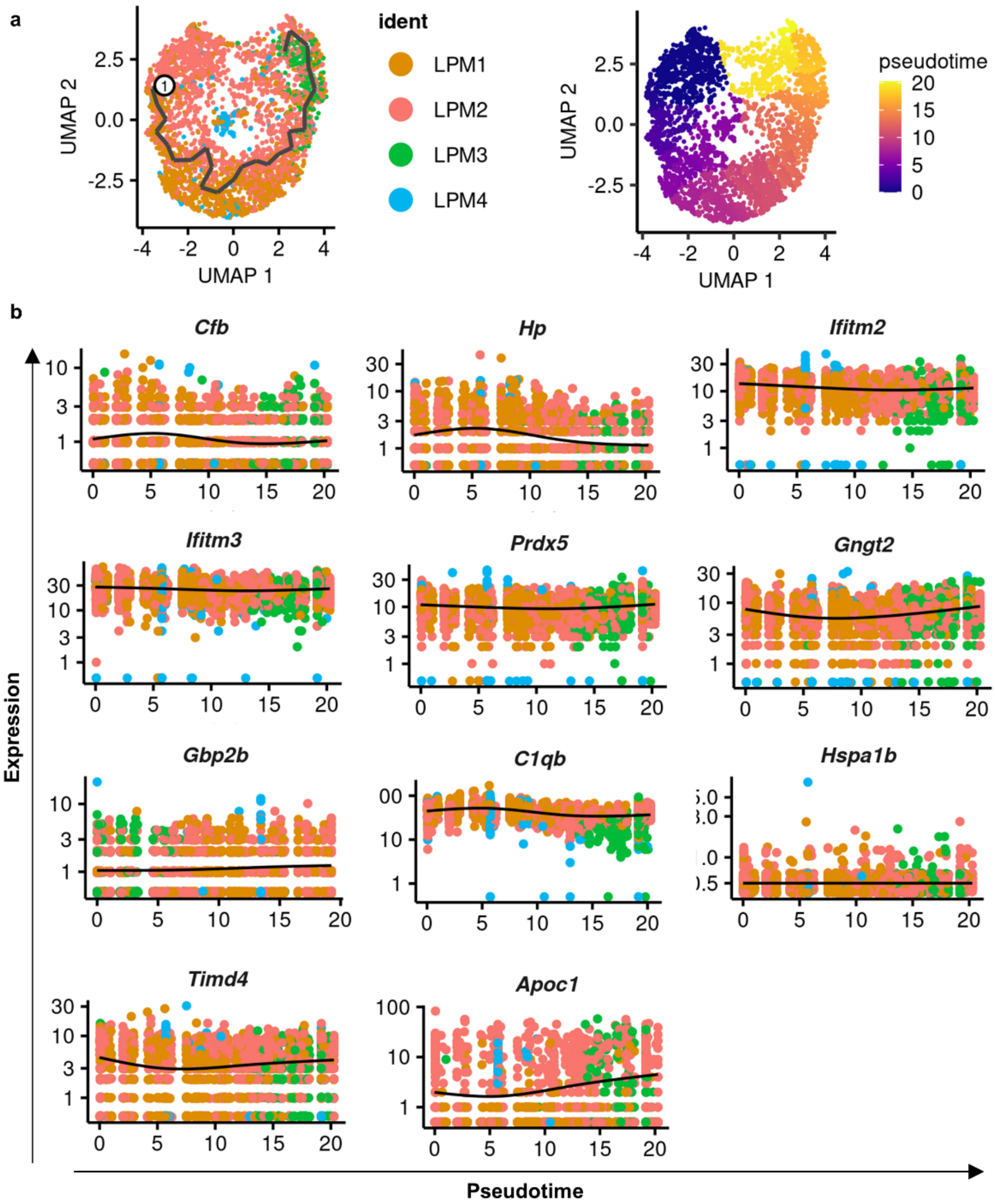

a UMAP showing the selected cells (LPM1-4) and the trajectory path built within them. The selected root cell was labeled as ①. **b** Dynamic expression of genes along this trajectory path. LPM1-4, *Timd4*$^{+}$ “large” peritoneal macrophage subtype 1-4.

Supplementary Fig. 7: Representative genes that were differentially expressed in patients with endometriosis.

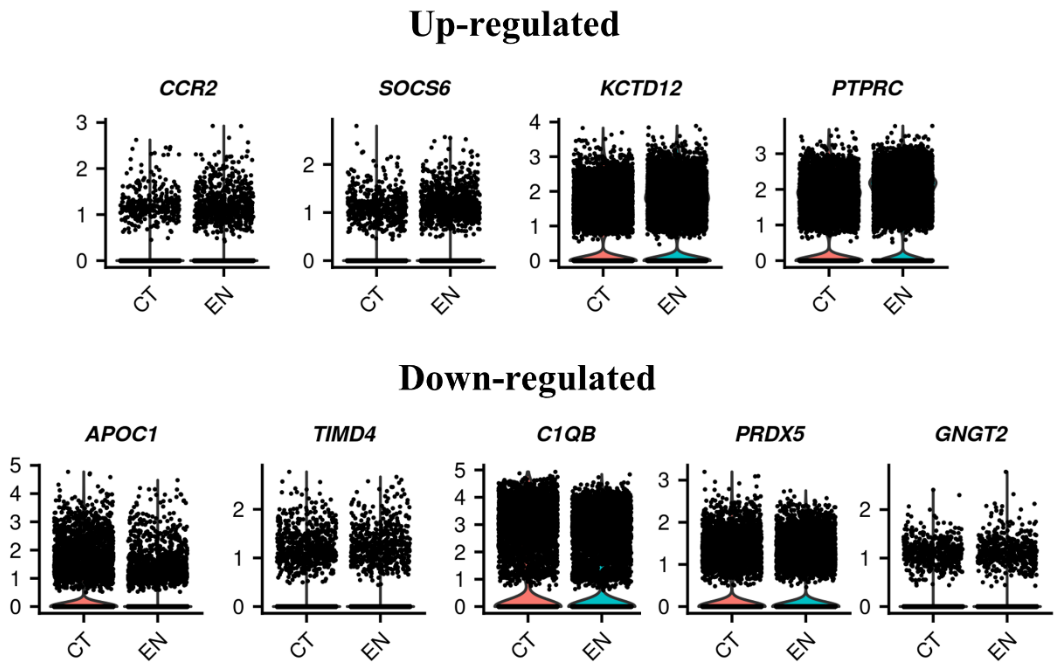

Publicly-available single-cell RNA-seq datasets of peritoneal exudate cells isolated from patients with or without endometriosis [SRR13962189, SRR13962190] were re-analyzed in this study. Raw data in FASTQ format were pre-processed with Cell Ranger V6.1.1 (10x Genomics), and sequencing depth between samples was normalized by cellrangerAggr (V6.1.1). The mean number of sequencing reads is 11,000. Gene-cell matrices were loaded into R using the Seurat package (V4.0.4) and processed following the standard pipeline. ITGAM (CD11b)$^{+}$ cell clusters were annotated as macrophages and were used for the analysis of differential gene expression (Wilcox test). All genes shown are statistically different ($p<0.05$).

Supplementary Fig. 8: Gating strategy for flow cytometry.

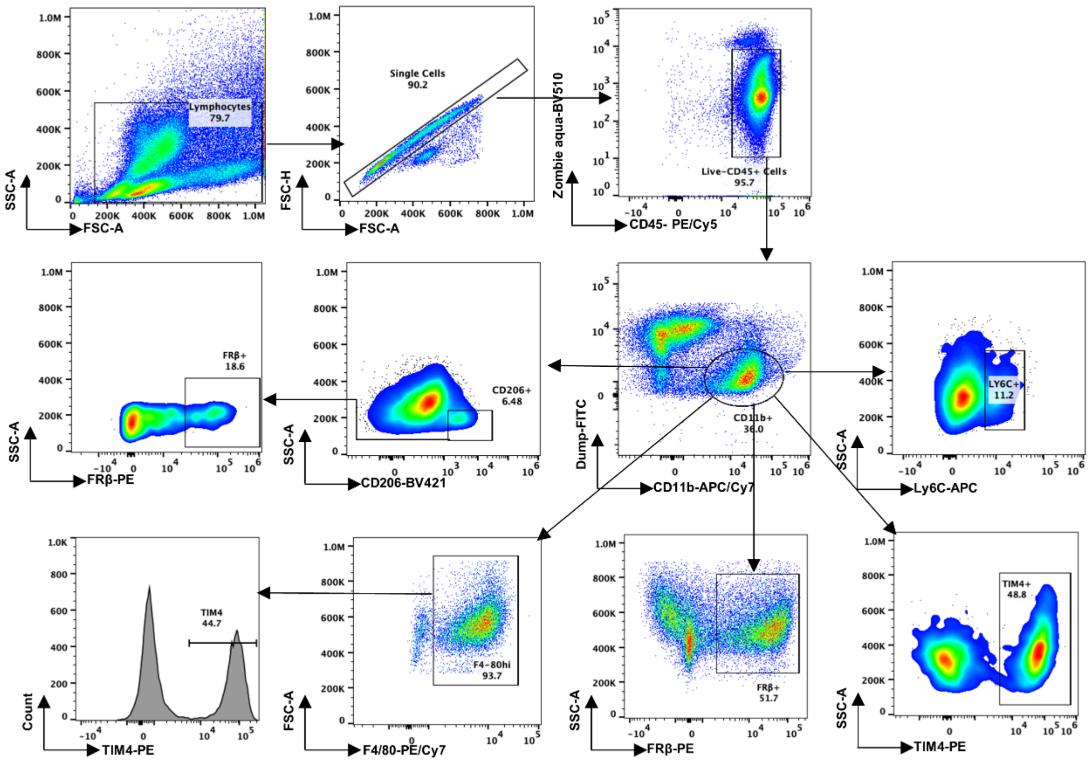

Supplementary Data Legends

Supplementary Data 1 Antibodies and reagents for Flow Cytometry.

Supplementary Data 2 Primer information used for RT-qPCR.

Supplementary Data 3 Enriched GO terms of biological processes in each macrophage subpopulation.

Supplementary Data 4 Enriched GSEA terms of biological processes in macrophages between treatments.

Supplementary Data 5 Genes and GO biological processes affected by in silico knockout of *Retnla*, *Cfb*, and *Timd4*.

Supplementary Data 6 Enriched GSEA terms of biological processes in B cells between treatments.
