# Supplementary material for: Niclosamide targets the dynamic progression of macrophages for the resolution of endometriosis in a mouse model
Source: Commun Biol. 2022 Nov 11;5:1225. doi: 10.1038/s42003-022-04211-0 (PMC9652344; doi:10.1038/s42003-022-04211-0)
Supplement: Supplementary file 3 — Description of Additional Supplementary Files [file 42003_2022_4211_MOESM3_ESM.pdf]

Description of Additional Supplementary Files

File name: Supplementary Data 1

Description: Antibodies and reagents for Flow Cytometry

File name: Supplementary Data 2

Description: Primer information used for RT-qPCR

File name: Supplementary Data 3

Description: Enriched GO terms of biological processes in each macrophage subpopulation (Pre-SPMs)

File name: Supplementary Data 4

Description: Enriched GSEA terms of biological processes in macrophages between treatments (overlaps of ELL/sham and ELL_N/ELL)

File name: Supplementary Data 5

Description: Genes and GO biological processes affected by in silico knockout of Retnla, Cfb and Timd4. (GO biological processes, Timd4 KO)

File name: Supplementary Data 6

Description: Enriched GSEA terms of biological processes in B cells between treatments (overlaps between ELL/sham and ELL_N/ELL)
