# Supplementary material for: Niclosamide targets the dynamic progression of macrophages for the resolution of endometriosis in a mouse model
Source: Commun Biol. 2022 Nov 11;5:1225. doi: 10.1038/s42003-022-04211-0 (PMC9652344; doi:10.1038/s42003-022-04211-0)
Supplement: Supplementary file 10 — Reporting Summary [file 42003_2022_4211_MOESM10_ESM.pdf]

nature portfolio

Corresponding author(s): Kanako Hayashi

Last updated by author(s): Sep 20, 2022

Reporting Summary

Nature Portfolio wishes to improve the reproducibility of the work that we publish. This form provides structure for consistency and transparency in reporting. For further information on Nature Portfolio policies, see our Editorial Policies and the Editorial Policy Checklist.

Statistics

For all statistical analyses, confirm that the following items are present in the figure legend, table legend, main text, or Methods section.

n/a	Confirmed	
☐	☒	The exact sample size (*n*) for each experimental group/condition, given as a discrete number and unit of measurement
☐	☒	A statement on whether measurements were taken from distinct samples or whether the same sample was measured repeatedly
☐	☒	The statistical test(s) used AND whether they are one- or two-sided<br>*Only common tests should be described solely by name; describe more complex techniques in the Methods section.*
☐	☒	A description of all covariates tested
☐	☒	A description of any assumptions or corrections, such as tests of normality and adjustment for multiple comparisons
☐	☒	A full description of the statistical parameters including central tendency (e.g. means) or other basic estimates (e.g. regression coefficient) AND variation (e.g. standard deviation) or associated estimates of uncertainty (e.g. confidence intervals)
☐	☒	For null hypothesis testing, the test statistic (e.g. *F*, *t*, *r*) with confidence intervals, effect sizes, degrees of freedom and *P* value noted<br>*Give P values as exact values whenever suitable.*
☒	☐	For Bayesian analysis, information on the choice of priors and Markov chain Monte Carlo settings
☒	☐	For hierarchical and complex designs, identification of the appropriate level for tests and full reporting of outcomes
☒	☐	Estimates of effect sizes (e.g. Cohen's *d*, Pearson's *r*), indicating how they were calculated

Our web collection on statistics for biologists contains articles on many of the points above.

Software and code

Policy information about availability of computer code

Data collection	Peritoneal exclude cells were collected and prepared for single-cell RNA sequencing following the manufacturer's protocol (10X Genomics, Inc.) of the Chromium Single Cell 3' Library & Gel Bead Kit V3.
Data analysis	Raw data in FASTQ format were preprocessed with Cell Ranger V3.1.0 (10x Genomics) mapping to the mouse GRCm38/mm10 transcriptome to generate gene-cell matrices. Then the gene matrix of three libraries were integrated into R using the Seurat package (V4.0.4) and processed following the standard pipeline of Seurat. Gene Ontology (GO) and Gene Set Enrichment Analysis (GSEA) was performed with the R package, clusterProfiler V3.18.0, using all detected genes from the entire scRNA-seq library as background. Pseudo-trajectory analyse were conducted using the package of Monocle 3 in R following the standard pipeline. Functional analysis of Retnla, Cfb, and Timd4 was conducted using the R package of scTenifoldKnk V1.0.1. Gene expression data of Seurat objects were used as input to model the probability of intercellular interactions between B cells and macrophages using the R package of CellChat V1.0.0.

For manuscripts utilizing custom algorithms or software that are central to the research but not yet described in published literature, software must be made available to editors and reviewers. We strongly encourage code deposition in a community repository (e.g. GitHub). See the Nature Portfolio guidelines for submitting code & software for further information.

Data

Policy information about availability of data

All manuscripts must include a data availability statement. This statement should provide the following information, where applicable:

- Accession codes, unique identifiers, or web links for publicly available datasets
- A description of any restrictions on data availability
- For clinical datasets or third party data, please ensure that the statement adheres to our policy

The scRNA-seq data are openly available in the GEO database at NCBI, reference number GSE147024.

Human research participants

Policy information about studies involving human research participants and Sex and Gender in Research.

Reporting on sex and gender	*Use the terms sex (biological attribute) and gender (shaped by social and cultural circumstances) carefully in order to avoid confusing both terms. Indicate if findings apply to only one sex or gender; describe whether sex and gender were considered in study design whether sex and/or gender was determined based on self-reporting or assigned and methods used. Provide in the source data disaggregated sex and gender data where this information has been collected, and consent has been obtained for sharing of individual-level data; provide overall numbers in this Reporting Summary. Please state if this information has not been collected. Report sex- and gender-based analyses where performed, justify reasons for lack of sex- and gender-based analysis.*
Population characteristics	*Describe the covariate-relevant population characteristics of the human research participants (e.g. age, genotypic information, past and current diagnosis and treatment categories). If you filled out the behavioural & social sciences study design questions and have nothing to add here, write "See above."*
Recruitment	*Describe how participants were recruited. Outline any potential self-selection bias or other biases that may be present and how these are likely to impact results.*
Ethics oversight	*Identify the organization(s) that approved the study protocol.*

Note that full information on the approval of the study protocol must also be provided in the manuscript.

Field-specific reporting

Please select the one below that is the best fit for your research. If you are not sure, read the appropriate sections before making your selection.

☒ Life sciences ☐ Behavioural & social sciences ☐ Ecological, evolutionary & environmental sciences

For a reference copy of the document with all sections, see nature.com/documents/nr-reporting-summary-flat.pdf

Life sciences study design

All studies must disclose on these points even when the disclosure is negative.

Sample size	Based on the standard deviations obtained from our previous studies (qPCR and Flow Cytometry), we conducted a power analysis and choose the sample size (n=6 for qPCR and n=5 for Flow Cytometry) that could provide sufficient power to discern treatment effects. The number of sinlge cells used for scRNAseq were based on previous studies.
Data exclusions	All data were included.
Replication	Some important findings from scRNA-seq were verified by qPCR and Flow Cytometry. Six biological replicates were used for qPCR and 5 for Flow Cytometry.
Randomization	Female mice at similar age were assigned to different groups randomly.
Blinding	During data collection, samples were partially blinded to the investigator.

Reporting for specific materials, systems and methods

We require information from authors about some types of materials, experimental systems and methods used in many studies. Here, indicate whether each material, system or method listed is relevant to your study. If you are not sure if a list item applies to your research, read the appropriate section before selecting a response.

Materials & experimental systems

n/a	Involved in the study
☐	☒ Antibodies
☒	☐ Eukaryotic cell lines
☒	☐ Palaeontology and archaeology
☐	☒ Animals and other organisms
☒	☐ Clinical data
☒	☐ Dual use research of concern

Methods

n/a	Involved in the study
☒	☐ ChIP-seq
☐	☒ Flow cytometry
☒	☐ MRI-based neuroimaging

Antibodies

Antibodies used

Antibodies used in this study were provided in Supplementary Data S1.
CD11b M1/70 APC-Cy7 BD Biosciences 557657 AB_396772
CD19 6D5 FITC BioLegend 115505 AB_313640
CD206 MMR BV421 BioLegend 141717 AB_2562232
CD209a MMD3 PE BioLegend 833003 AB_2721636
CD3 17A2 FITC BioLegend 100203 AB_312660
CD335 29A1.4 FITC BioLegend 137605 AB_2149150
CD45 30-f11 PE-Cy5 BioLegend 103109 AB_312974
FRβ 10/FR2 PE BioLegend 153303 AB_2721343
Siglec-F/CD170 S17007L FITC BioLegend 155503 AB_2750232
Ly6C AL-21 APC BD Biosciences 560595 AB_1727554
Ly6G/Ly6C/Gr-1 RB6-8C5 FITC BioLegend 108405 AB_313370
TIM4 RMT4-54 PE BioLegend 130005 AB_1227807
Fc Block CD16/CD32 antibody Thermo Fisher 14-0161-82 AB_467133
Total Antibody Compensation Bead Kit Thermo Fisher A10513
Zombie Aqua™ Fixable Viability Kit BioLegend 423101

Validation

Antibodies were validated by the manufacturers and our previous publications.

Animals and other research organisms

Policy information about studies involving animals; ARRIVE guidelines recommended for reporting animal research, and Sex and Gender in Research

Laboratory animals

C57BL/6J mice were purchased from the Jackson Laboratory

Wild animals

No wild animals were used.

Reporting on sex

Only female mice were used in this study as endometriosis is a disease for women

Field-collected samples

No field-collected samples were used

Ethics oversight

All procedures were performed in accordance with the guidelines approved by the Institutional Animal Care and Use Committee of the Washington State University (Protocol # 6751).

Note that full information on the approval of the study protocol must also be provided in the manuscript.

Flow Cytometry

Plots

Confirm that:

☒ The axis labels state the marker and fluorochrome used (e.g. CD4-FITC).

☒ The axis scales are clearly visible. Include numbers along axes only for bottom left plot of group (a 'group' is an analysis of identical markers).

☒ All plots are contour plots with outliers or pseudocolor plots.

☒ A numerical value for number of cells or percentage (with statistics) is provided.

Methodology

Sample preparation

Peritoneal exudate cells from three mice were pooled as one sample and used for analysing immune cell profiles by flow cytometry. A total of 15 mice were used for each group (n=5). Briefly, the peritoneal lavages were centrifuged to collect peritoneal exudate cells. After lysing red blood cells by 1x RBC Lysis Buffer (BioLegend), an equal number of cells from each

group were incubated at room temperature for 20 minutes with Zombie Aqua™ Fixable Viability dye (BioLegend) and blocked on ice for 20 minutes with Fc Block anti-CD16/CD32 (Thermo Fisher). Then cells were stained with fluorochrome-conjugated monoclonal antibodies (Data S1) for 1 hour.

Instrument

Samples were acquired with the Attune NxT Acoustic Focusing Cytometer using Attune NxT software (Thermo Fisher)

Software

Data were analysed with FlowJo v10.4.

Cell population abundance

Each cell population was identified using its specific protein markers and assessed with intensity. The purity of samples was achieved by using only single and live cells for analysis (doublets and clumps of cells were excluded), and by dumping the irrelevant cell populations (e.g., T and B cells) from macrophages.

Gating strategy

The level of background signal was determined by using unstained cells mixed from all three experimental groups. Fluorescence compensation was calculated via single-stained controls. Scatter profiles were first used to select single cells, then live, and leukocytes (CD45 positive) were gated. Lineages including T, B, NK cells, and eosinophils were defined by CD3, CD19, CD335, and Siglecs-F positive populations, respectively, and eliminated from further analysis. Total macrophages were identified by CD11b+ cells and were used for determining the subpopulations. CD206+, FRbeta+, Ly6C+, F4/80+, and TIM4+ macrophages were selected for cells that show strong fluorescence indicating their specific proteins.

☒ Tick this box to confirm that a figure exemplifying the gating strategy is provided in the Supplementary Information.
